# Detection of gynaecological cancer in pregnancy

**DOI:** 10.1186/1470-7330-15-S1-P34

**Published:** 2015-10-02

**Authors:** F Cuthbert, N Bharwani, A Rockall

**Affiliations:** 1 Brighton and Sussex University Hospital, 177 Preston Road, Brighton, BN1 6AG, UK

## Learning objectives

The purpose of this educational poster is to:

• Review the challenges and limitations of imaging gynaecological cancer in pregnancy

• To illustrate that optimizing imaging can provide diagnostically useful information to the multidisciplinary team, in particular we will review the principles and protocols of MR imaging in this context

• Review the imaging appearances of gynaecological cancer in pregnancy

## Content organisation

When a pregnant patient presents with gynaecological cancer several issues must be considered by the multidisciplinary team – disease stage, nodal status, gestational age, obstetric complications and, importantly, the patient’s wishes regarding treatment where a balance must be sought between foetal well-being and optimal maternal therapy.

As always, diagnostic imaging is key to these decisions but in pregnant patients we are limited, we avoid CT and contrast agents and therefore ultrasound and MRI become paramount. We are further limited when diagnostic image quality is reduced by foetal movement artefact. We suggest and justify MR protocols written to maximise diagnostic yield.

We will present a narrative on the incidence and presentation of cervical cancer and ovarian cancer in pregnancy followed by a pictorial description of their key imaging findings at various disease stages. In the context of cervical cancer we will discuss considerations post fertility-preserving surgery.

## Conclusion

This educational poster will highlight the issues around imaging pregnant patients with gynaecological cancer, illustrate their imaging appearances and demonstrate the usefulness of MR imaging in this context.

